# Integrating DNA methylation and gene expression data in a single gene network using the iNETgrate package

**DOI:** 10.1038/s41598-023-48237-8

**Published:** 2023-12-08

**Authors:** Sogand Sajedi, Ghazal Ebrahimi, Raheleh Roudi, Isha Mehta, Amirreza Heshmat, Hanie Samimi, Shiva Kazempour, Aamir Zainulabadeen, Thomas Roderick Docking, Sukeshi Patel Arora, Francisco Cigarroa, Sudha Seshadri, Aly Karsan, Habil Zare

**Affiliations:** 1 Department of Cell Systems & Anatomy, The University of Texas Health Science Center, San Antonio, TX 78229 USA; 2 Glenn Biggs Institute for Alzheimer’s & Neurodegenerative Diseases, San Antonio, TX 78229 USA; 3 Bioinformatics Program, The University of British Columbia, Vancouver, BC Canada; 4 Department of Radiology, Stanford University School of Medicine, Stanford, CA 94305 USA; 5 Department of Immunology, University of Pittsburgh, Pittsburgh, PA 15213 USA; 6 Department of Imaging Physics, The University of Texas MD Anderson Cancer Center, Houston, TX 77030 USA; 7 School of Architecture, University of Utah, Salt Lake City, UT 84112 USA; 8 Department of Computer Science, Princeton University, Princeton, NJ 08540 USA; 9 Canada’s Michael Smith Genome Sciences Centre, British Columbia Cancer Research Centre, Vancouver, BC V5Z 1L3 Canada; 10 Mays Cancer Center, The University of Texas Health Science Center, San Antonio, TX 78229 USA; 11 Malu and Carlos Alvarez Center for Transplantation, Hepatobiliary Surgery and Innovation, The University of Texas Health Science Center, San Antonio, TX 78229 USA; 12 Department of Neurology, University of Texas, San Antonio, TX 78229 USA; 13 Department of Neurology, Boston University School of Medicine, Boston, Massachusetts 02139 USA; 14 Department of Cell Systems & Anatomy, 7703 Floyd Curl Drive, San Antonio, TX 78229 USA

**Keywords:** Computational models, Data integration, Data processing, Machine learning, Network topology, Computational biology and bioinformatics, Software

## Abstract

Analyzing different omics data types independently is often too restrictive to allow for detection of subtle, but consistent, variations that are coherently supported based upon different assays. Integrating multi-omics data in one model can increase statistical power. However, designing such a model is challenging because different omics are measured at different levels. We developed the iNETgrate package (https://bioconductor.org/packages/iNETgrate/) that efficiently integrates transcriptome and DNA methylation data in a single gene network. Applying iNETgrate on five independent datasets improved prognostication compared to common clinical gold standards and a patient similarity network approach.

## Introduction

Orthogonal data types, and specifically genomic and epigenomic profiles, can potentially provide new opportunities to pinpoint underlying molecular mechanisms of diseases^1^.

Approaches, which involve analysis of each data type independently, are often too conservative, as they would not allow for detection of subtle, but consistent, variations that would be supported based upon results from the independent assays.

New advanced biomedical informatics approaches are *critically needed* in which different data sets can be seamlessly and efficiently incorporated into a single comprehensive analysis.

Complex multi-omics data, including transcriptomics, epigenomics, and proteomics data, can be integrated using a network analysis approach^1–7^.

DNA methylation is essential for initiating gene expression and numerous cellular functions as an activation mark, however, abnormalities such as hypomethylation and hypermethylation at specific loci can contribute to the initiation and development of cancer^8^. Multiple methods have been developed to incorporate gene expression and DNA methylation data^9–13^.

For example, a similarity network fusion (SNF)^14^ approach can be used to identify similar patient subgroups in a patient similarity network^15,16^. In their approach, nodes represent individual patients and an edge corresponds to the similarity between two patients computed based on all available features.

While patient similarity networks identify patterns associated with complex data, biological interpretation of these patterns remains a significant challenge. Particularly, a deeper understanding of underlying molecular mechanisms, deregulated pathways, and interconnected variables is often implausible from such networks.

To overcome the complexities of integrative network analysis, we developed iNETgrate, a unified network where each node represents a gene, and an edge between a pair of genes is weighted based on both DNA methylation and gene expression data. In this way, iNETgrate incorporates DNA methylation and gene expression data into a unified network. This innovative paradigm employs a multi-view approach^17^ that enhances our previously established method, Pigengene^18^.

**Figure 1:**
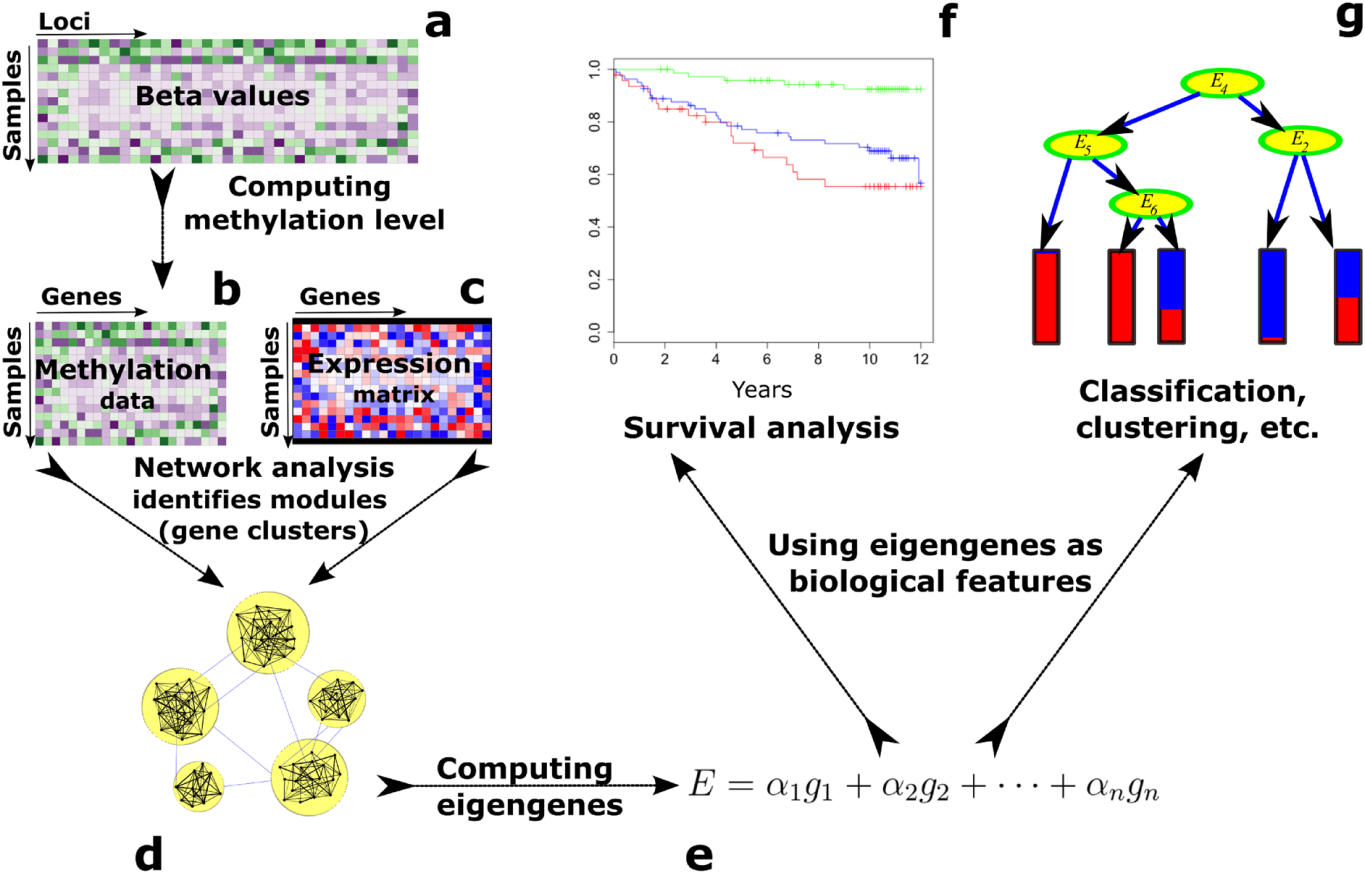
Schematic view of the methodology. The inputs include (**a**) a DNA methylation profile measured at genomic loci, which we use to compute (**b**) methylation value at the gene level, and (**c**) a gene expression profile. (**d**) We construct an integrative network, in which nodes represent genes and edges model the association between individual gene pairs based on both expression and methylation data (Eq. (1) in “Methods”). (**e**) For each module, we compute eigengenes as weighted averages of the expression and DNA methylation level of all genes in that module (Eqs. (2)–(4) in “Methods”). (**f**) We employ the eigengenes as robust biological signatures (i.e., biomarkers) for survival analysis. (**g**) While not implemented in this study, the eigengenes could also be utilized for other downstream data mining analyses.

The iNETgrate framework (Fig. 1) starts with preprocessing the available data (Methods). Then, we compute a DNA methylation value for each gene. This is a key step in the iNETgrate workflow because it results in every node (i.e., gene) in our network having two features, namely, gene expression and DNA methylation levels. To quantify the DNA methylation level associated with a gene, iNETgrate computes a weighted average of the corresponding beta values using a principal component analysis^19^ (PCA). Specifically, the first principal component is computed, which we call an *eigenloci* in our paradigm, and used to represent the loci at the gene level. When the number of loci corresponding to a gene is more than a threshold, a subset of them is used as detailed in the “Methods”.

The iNETgrate computes the weight of an edge between a pair of genes in three steps: (a) correlation based on DNA methylation at the gene level and (b) correlation based on gene expression are computed, then, (c) the absolute correlations are combined with an integrative factor of $\mu$ (Eq. (1) in “Methods”). We then use a refined hierarchical clustering method^20^ to identify gene modules, where each module is a cluster of similar genes based on both gene expression and DNA methylation data.

An eigengene is the first principal component of the data in a module. For each gene module, we use PCA to compute two eigengenes, where each eigengene is a weighted average of gene expression level, DNA methylation levels, or both for the genes in the corresponding module (Eqs. (2), (3), (4) in “Methods”, respectively). Eigengenes are robust biological features useful for downstream data mining analyses e.g., classification^18^, survival analysis^21^, and prognostication^1^. Here, we illustrate the application of eigengenes in determining risk groups in different diseases and show the advantage of integrating DNA methylation data in a gene co-expression network.

We benchmarked iNETgrate against two other methodologies using five independent datasets including four cohorts from The Cancer Genome Atlas(TCGA): lung squamous carcinoma (LUSC)^22^, lung adenocarcinoma (LUAD)^23^, liver hepatocellular carcinoma (LIHC)^24^, and acute myeloid leukemia (AML)^25^. In addition, we used a cohort from the Religious Orders Study^26^ and Memory and Aging Project^27,28^ (ROSMAP) including cases with different stages of Alzheimer’s Disease and Related Dementias (ADRD).

We compared the iNETgrate performance in identifying risk groups with (a) clinical gold standards within each cohort and (b) a well-known similarity network tool called the Similarity Network Fusion tool^14^ (SNFtool). Unlike the iNETgrate approach, SNFtool is based on the similarity between the subjects (i.e., patients), and not the genes. The SNFtool first computes a similarity matrix using each data type (i.e., view) such as gene expression and DNA methylation. Then, the similarity matrices are fused into a network, where each node represents a patient and connections are established between two patients based on the fused similarity patterns.

## Results

For a clearer presentation, we only discuss the outcomes for LUSC here and report results on the other four datasets in the supplementary materials (Supplementary Fig. S2).

We assigned different values for $\mu$ in Eq. (1) (“Methods”) from 0, which results in using only the gene expression data, to 1, which results in using only DNA methylation data, with a 0.1 increment. The best performing $\mu$ for our survival analysis in the LUSC cohort was $\mu =0.4$.

We identified 71 gene modules (i.e., clusters) from our integrated network. We computed two eigengenes for each module using the DNA methylation at the gene level (suffixed with “m”) and the gene expression (suffixed with “e”) data. We also computed a linear combination of these two eigengenes (suffixed with “em”) using coefficients $\mu =0.4$ and $1-\mu =0.6$, respectively. We used a penalized Cox regression model^29,30^ to determine the best subset of three eigengenes out of the $3*71=213$ available eigengenes. We found that the most associated subset of three eigengenes with overall survival included eigengenes 23 m, 64 m, and 44 em. Next, we employed an accelerated failure time (AFT) model^31^ to determine the optimal combination from the three selected eigengenes for predicting survival time, which revealed that eigengenes 23 m and 64 m make the best model for predicting survival in this dataset.

**Figure 2:**
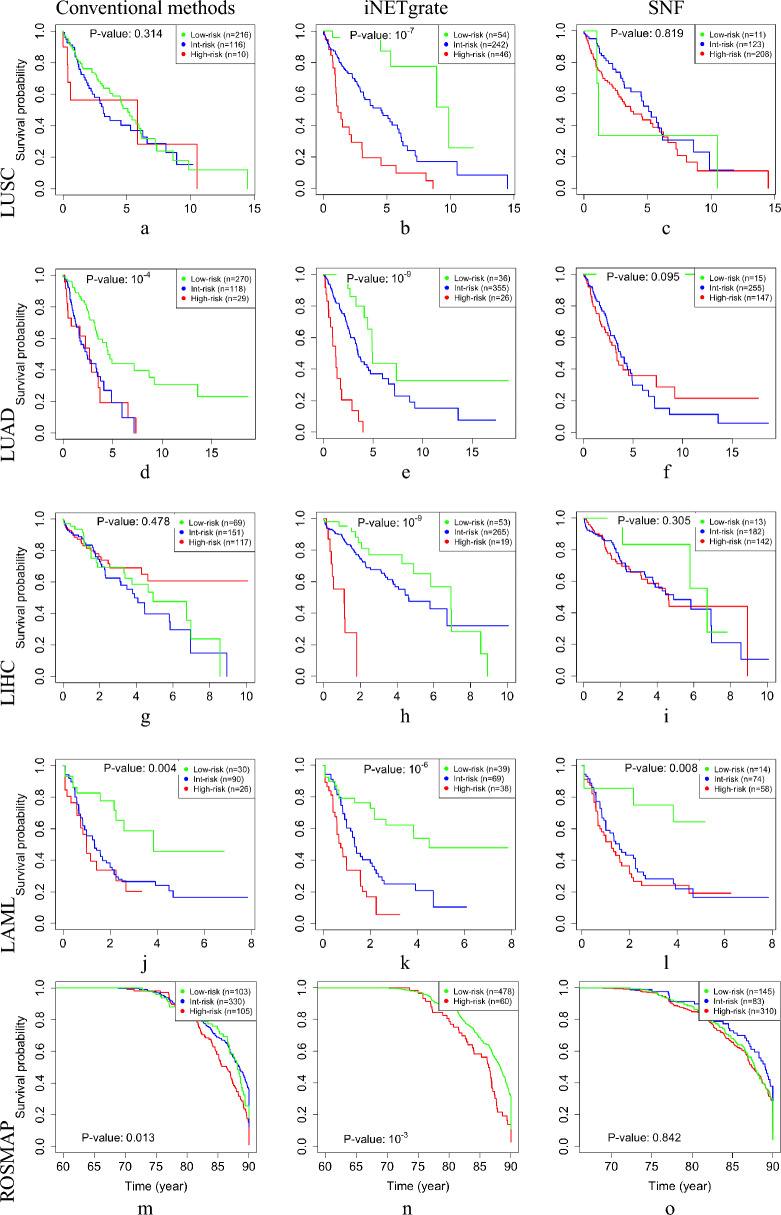
The Kaplan–Meier^49^(KM) curves for all dataset. The log-rank $p$–values indicate that differences between the low-risk group (green) and the high-risk group (red) using clinical criteria (**a, d, g, j, m**), iNETgrate (**b, e, h, k, n**), and SNFtool (**c, f, i, l, o**). In all datasets (on rows), using iNETgrate, the middle column, resulted in a significantly smaller p-values compared with the conventional classification methods in clinics (left column) and an integrative network method of SNFtool (right column).

Using this AFT model^31^ with 23 m and 64 m, we categorized the patients into three groups of 54 low-, 242 intermediate-, and 46 high-risk patients (Fig. 2b). The high-risk group identified by iNETgrate had a significantly shorter survival time than the low-risk group (p-value $\le 10^{-7}$, Table 1). This is a major improvement over the stratification by clinical gold standards (Fig. 2a, p-value 0.314) and the state-of-the-art SNFtool in this dataset (Fig. 2c, p-value 0.819).

In all five studied datasets, the survival analyses based on the eigengenes provided by iNETgrate resulted in the best p-values in the range of $10^{-9}$ to $10^{-3}$ (Fig. 2 and Table 1), whereas SNFtool and the clinical gold standard led to p-values less than 0.01 in only one and two datasets, respectively.

To understand the genomic and epigenomic landscape associated with survival outcomes, we investigated the individual contributions of DNA methylation and gene expression data. Analyzing each modality individually, (i.e., making models based solely on gene expression using $\mu =0$ or DNA methylation using $\mu =1$) resulted in a p-value of $10^{-4}$. Whereas, optimizing the integrative factor to $\mu =0.4$ generated a relatively more significant p-value of $10^{-7}$. This finding underscores the power of our multi-omics integration strategy in capturing a holistic representation, thereby, substantially improving the prognostic prediction capabilities of the survival model.

Furthermore, different cohorts of the same disease can be readily merged because correlations computed based on different datasets can be easily combined and used in the network. We compared the computational performance of the iNETgrate method with SNFtool. While SNFtool completes its analyses in a couple of minutes, iNETgrate requires longer computational time of around 6 h to analyze the same data. Although speed is an advantage for SNFtool, it fails to convey the complete perspective. In particular, iNETgrate consistently yields significant p-values for the prognostication of risk groups, indicating higher precision and more efficient use of biological information in the multi-omics data compared to SNFtool. Gene modules identified by iNETgrate can be investigated in different ways including pathway enrichment analysis, hub gene identification, and analysis of gene weights based on eigengenes among others. These downstream analyses are essential for biological interpretation of multi-omics data and obtaining a comprehensive view of underlying molecular mechanisms. In contrast, patient similarity networks provide limited information on why cases are grouped together.

Using the Kyoto Encyclopedia of Genes and Genomes (KEGG)^32–36^, our pathway analysis on the selected modules in the LUSC dataset revealed a significant association with a total of 15 genes that were enriched in four pathways: the neuroactive ligand–receptor interaction, the cAMP signaling, the calcium signaling, and the glutamatergic synapse pathways. These pathways are known to be related to LUSC as detailed below.

Our observation of an association between the cAMP signaling pathway and LUSC was previously reported by Zhang et al., who identified the GRM8 signaling pathway as a potential therapeutic target for squamous cell lung cancer^37^. The connection between GRM8 and cAMP is crucial, as the activation of GRM8 can modulate adenylate cyclase activity, impacting the cAMP signaling pathway. The research by Wen et al. outlines how smoking-activated signaling pathways, including the cAMP signaling pathway, play key roles in lung carcinogenesis, particularly in LUSC^38^. Furthermore, the calcium signaling pathway as a potential key in the context of LUSC was previously substantiated by Ke et al.^39^ They demonstrated that miR-147b has differentially expressed genes significantly associated with the calcium signaling pathway in LUSC, which is crucial for several cellular processes, including drug transport and DNA binding.

Previous studies corroborate the association between the neuroactive ligand–receptor interaction pathway and LUSC through extensive analysis among LUSC patients and controls^40,41^. The Glutamatergic synapse pathway occurred as a significant pathway concerning LUSC. This outcome aligns with a previous study by Zhang et al., which also highlighted an association between LUSC and the Glutamatergic synapse pathway, supporting the potential relevance of this pathway in the context of LUSC^42^.

We undertook a bootstrap analysis on the LUSC dataset to investigate the robustness of iNETgrate and particularly, to evaluate the potential effects of outliers on the stability of our results. Bootstrap is a resampling technique that provides empirical evidence on the strength of statistical estimates^43^. We applied bootstrap sampling three times with 100, 500, and 1000 iterations, respectively. Our experiments across these samplings presented remarkable consistency. Specifically, at $\mu =0.4$, which was the best value based on our original results, the mean of p-values remained significant and stable at around $10^{-4}$ across the three bootstrap samplings, with relatively small variances of 0.000, 4, 0.000, 7, and 0.000, 6 for the 100, 500, and 1000 iterations, respectively. This implies that our model is resilient to potential outliers and random variations. The relatively more significant p-value from our original experiment without bootstrapping is justified by having more unique patients compared to a bootstrap sample.

## Discussion

Our experiments collectively show that integrating DNA methylation and gene expression in a single gene network increases statistical power. The rationale for integrating DNA methylation in our iNETgrate analysis is that DNA methylation, as an epigenetic modification, plays a crucial role in gene regulation. Observing co–methylation patterns, mainly among genes in close genomic proximity, usually reveals shared regulatory elements or similar chromatin environments^44,45^. These patterns act as indicators of genomic regions under corresponding regulatory effects. While this could naturally cluster genes together due to shared patterns, it is crucial to identify and account for the inherent spatial bias, where neighboring genes may exhibit co-methylation merely due to their genomic positioning. By incorporating gene expression and DNA methylation data using iNETgrate, we ensure our approach is not solely reliant on methylation patterns.

Among the current approaches for integration of epigenome and transcriptome data, iNETgrate is unique in that it can include the available information from all genes in a single gene network. Some alternative methods are described below. A notable study by Ren et al. presents a network-based framework, especially suitable when dealing with skewed survival time data prone to outliers^46^. Their method uniquely employs a weighted least absolute deviation objective function and develops a network-based variable selection method using the AFT model. However, when contrasting with iNETgrate, fundamental differences arise. iNETgrate incorporates a broader spectrum of genes, ensuring wide recognition of potentially significant genes from the entire gene set, unlike the Ren et al. selective approach that includes only a couple of hundreds of genes. Furthermore, iNETgrate integrates DNA methylation and gene expression data, providing a multi-omics perspective, which could account for the relatively higher accuracy of survival estimates.

Zachariou et al.^47^ introduced an approach for integrating six different types of interactions to identify significant pathways related to a disease using a “super network”. Their method then performed pathway analysis on top genes based on the quantity of shared information between gene pairs. However, it is not clear how DNA methylation can be included in the construction of their network. In contrast, iNETgrate incorporates DNA methylation data and expands the depth of information in the integrated network, which potentially provides more holistic insight into gene interactions and the corresponding regulatory mechanisms. Moreover, iNETgrate builds a comprehensive gene–level network, discovering complex details about gene–gene relationships that might be overlooked in pathway-focused analyses.

Edge-Based Module Detection Network (EMDN)^48^ is another integrative approach at the gene level. In this approach, differential co-methylation and co-expression networks are first constructed, then the standard modules within multiple networks are defined as epigenetic modules. While EMDN’s capacity to identify and focus on differentially expressed and methylated genes allows for the elucidation of critical changes associated with disease states, it inherently limits the scope of the investigation to these selected genes and methylation sites. Consequently, other potential molecular interactions and gene modifications that do not reach the defined differential expression or methylation threshold are neglected, potentially leading to losing critical biological information.

Another limitation of EMDN and similar methods that rely on differential expression analysis is their assumption of having a case–control labeling in datasets, which limits their application in research settings such as survival or clustering analyses where matched data are not readily available. These considerations highlight the added value of iNETgrate, which is more inclusive and is designed to utilize all available gene and methylation data rather than limiting the analysis to only differentially expressed or differentially methylated features. In this way, iNETgrate can use the subtle, but constant, variations in the data that might be missed by any approach that starts with a differential analysis. Additionally, the flexibility of iNETgrate to work efficiently without the need for matched control data emphasizes its usefulness in a broader range of research applications.

**Table 1:** Comparison of conventional clinical methods, SNF, and iNETgrate.

Datasets	Conventional methods		SNFtool			iNETgrate		
	Criteria	p-value	Genes	Loci	p-value	Genes	Loci	p-value
LUSC	Pathologic	0.314	12,231	89,213	0.819	12,494	239,703	$10^{-7}$
LUAD	Pathologic	$10^{-4}$	7362	49,515	0.095	7535	165,478	$10^{-9}$
LIHC	AFP & Ishak score	0.478	12,198	112,398	0.305	13,239	240,905	$10^{-9}$
LAML	Cytogenetics	0.004	9677	71,022	0.008	10,488	213,255	$10^{-6}$
ROSMAP	Braak	0.013	4942	484	0.709	11,646	240,021	$10^{-3}$

## Methods

### Description of datasets

In this study we, utilized five independent cohorts including four cancer- and one Alzheimer-related datasets. Gene expression profiling was done using RNA-seq and DNA methylation data were obtained using the Illumina Infinium HumanMethylation450 BeadChip, measuring DNA methylation levels (beta values) on about 450,000 genomic loci.

The TCGA cohorts were obtained using the TCGAbiolinks package^50^ (Version 2.24.3). TCGA-LUSC^22^ and TCGA-LUAD^23^ had clinical and genomic data from 589 and 592 patients, respectively (Supplementary Table S2). Information on the pathological stages of the tumors was included in both datasets. We used this information to stratify the patients into distinct risk groups and compared the resulting stratification with clusters obtained from our approach.

TCGA-LIHC^24^ was provided by a comprehensive study that included 436 cases with clinical information available in the data. We used the Ishak fibrosis score^51^ and alpha-fetoprotein (AFP) level^52–56^ to stratify patients into low-, intermediate-, and high-risk groups. The employed score is described later in this section.

TCGA-L AML was provided by a thorough genomic and epigenomic study on 200 adult cases with AML^25^. The risk groups were defined based on cytogenetic abnormalities^25,57^.

In addition, we used the ROSMAP cohort provided by the longitudinal cohort studies of aging and dementia. We downloaded the ROSMAP dataset from accelerating Medicines Partnership- AD^58^ with Synapse IDs syn3388564 (bulk RNA-seq) and syn5850422 (DNA methylation), using the synapser (https://r-docs.synapse.org/articles/synapser.html) R package (Version 0.6.61) and a custom R scripts (Version 3.6.1)^59^.

In the TCGA cohorts, events were defined by patients’ death and the time to an event referred to the duration from the initial diagnosis to death time or the last follow-up. In the ROSMAP cohort, the event was clinical diagnosis of any dementia including mild cognitive impairment with or without other cognitive conditions, Alzheimer’s dementia with or without other cognitive conditions, and other primary causes of dementia without clinical evidence of Alzheimer’s dementia. The time to an event in this context referred to the age at which the first dementia–related diagnosis was made.

To enhance the power of our network, we included cases that have either a single type of data (i.e., gene expression or DNA methylation) or both data available. In the survival analysis, we included only patients whose gene expression, DNA methylation, and survival data were available (Supplementary Table S2).

### Preprocessing data

The initial step in preprocessing involves normalizing the gene expression data. This is accomplished via a logarithmic transformation in based 10 to stabilize the variance and make the data more amenable to following analyses. Because logarithm of zero is not defined, a small offset is added to the expression levels prior to applying this transformation. iNETgrate further preprocesses data in two steps: cleaning and filtering. The former step involved cleaning DNA methylation and clinical data using the wrapper function cleanAllData(). Loci with more than $50\%$ missing beta values were removed, while loci with less than $50\%$ missing values were imputed. The imputation was performed by replacing each missing value with the mean of the beta values for the corresponding locus (preprocessDnam()). The clinical data was subsequently cleaned by removing cases with missing survival time and status (prepareSurvival()). The cleaned survival data had patient information including ID, events, time, and risk based on the clinical gold standard.

The second step in the preprocessing data was filtering out genes and loci that have a weak absolute Pearson correlation with survival time and vital status. This was performed by calling electGenes() inside the cleanAllData() wrapper function. In this study, we set the absolute correlation coefficient cutoffs to 0.2 in all TCGA datasets and 0.1 in the ROSMAP dataset.

Every gene and locus that met the quality control criteria was retained for the subsequent steps. In addition, we used computeUnion() to include corresponding loci of the selected genes and corresponding genes of the selected loci in the next steps of analysis.

### Calculating DNA methylation levels for genes

In iNETgrate, every node represents a gene with two features (i.e., gene expression and DNA methylation values). Therefore, we needed to calculate the DNA methylation value for each gene using computEigenloci(). This function calculated a weighted average of loci levels for their corresponding gene in the following way. When the number of loci corresponding to a gene was less than six, the first principal component (i.e., eigenloci) was calculated directly by taking a weighted average of beta values using PCA. This was the case for almost $95\%$ of loci in our datasets (Supplementary Fig. S1).

For the remaining $5\%$ of cases, in which the number of loci representing a gene was six or more, we used findCore() to determine the most connected cluster of loci for each gene. Specifically, a graph was constructed for each gene using the igraph package (Version 1.5.0). In this graph, each locus is represented by a node. We used a fast greedy algorithm^60^ to calculate the pairwise correlation between loci and detected communities (i.e., clusters) in the graph. Within each community, the average pairwise correlation was computed. The community with the highest average pairwise correlation was identified as a dense subset of highly co-methylated loci in the graph, and the eigenloci value was then computed based on this subset.

### Network construction and module detection

We constructed a network in which nodes represent genes and edges are weighted based on the absolute correlation of gene expression and DNA methylation levels for each pair of genes. This was achieved using the makeNetwork() function. The weight of the edges between genes $g_i$ and $g_j$ was calculated using the following equation:

1 $$\begin{aligned} \mathscr {W}(g_i,g_j)=(1-\mu )|{{\,\textrm{cor}\,}}_E(g_i,g_j)|+ \mu |{{\,\textrm{cor}\,}}_M(g_i,g_j)|, \end{aligned}$$

Here, $\mathscr {W}(g_i,g_j)$ describes the integrated similarity between genes $g_i$ and $g_j$. The term $|{{\,\textrm{cor}\,}}_E(g_i,g_j)|$ represents the absolute value of the Pearson correlation between the gene expression levels of genes $g_i$ and $g_j$. Similarly, $|{{\,\textrm{cor}\,}}_M(g_i,g_j)|$ represents the absolute value of the Pearson correlation between the DNA methylation levels of these two genes. The hyperparameter $\mu$ is an integrative factor controlling the relative contributions of gene expression and DNA methylation data in the network. When $\mu =0$, the network is based solely on gene expression data. Increasing the value of $\mu$ emphasizes the DNA methylation data in the model, whereas $\mu =1$ indicates that only DNA methylation data is used in calculating the edge weights (i.e., gene similarities).

Construction of the network and identification of the modules were done by the wrapper function makeNetwork(), which first uses the pickSoftTreshold() function (RsquaredCut=0.75) from the weighted gene co-expression network analysis^20^(WGCNA) package (Version 1.72.1) to determine the optimal soft-thresholding power for our integrated network. Then, the blockwiseModules() function (with minModuleSize=5, the absolute value of Pearson correlation, and the default values for the rest of parameters) is utilized to execute a hierarchical clustering approach. This leads to identification of modules, where each module is a group of genes that exhibit similar patterns of expression and DNA methylation. Additionally, module zero is designed to contain outlier genes that cannot be confidently assigned to any module due to their weak or negligible correlation with other genes.

### Module eigengene computation

We employed PCA to compute an eigengene for every module (computEgengenes()). In order to balance the contribution of high-risk and low-risk groups, the gene expression and DNA methylation data were oversampled. Intermediate-risk cases were not included in the PCA. An eigengene is computed from a weighted average of gene expression levels ($E^e$), DNA methylation levels ($E^m$), or both ($E^{em}$), using the following equations:

2 $$\begin{aligned} E^e = \alpha ^e_{_1} g^e_{_1} + \alpha ^e_{_2} g^e_{_2} + \cdots + \alpha ^e_{_n} g^e_{_n}, \end{aligned}$$

3 $$\begin{aligned} E^m = \alpha ^m_{_1} g^m_{_1} + \alpha ^m_{_2} g^m_{_2} + \cdots + \alpha ^m_{_n} g^m_{_n}, \end{aligned}$$

4 $$\begin{aligned} E^{em} = (1-\mu ) E^e + \mu E^m. \end{aligned}$$

Here, *n* is the number of genes in the module, $g^e_{_i}$ is the expression level of gene *i*, and $g^m_{_i}$ is the methylation level corresponding to gene *i* (i.e., eigenloci), while $\alpha ^e_{_n}$ and $\alpha ^m_{_n}$ are the corresponding weights. These weights are computed using PCA ensuring maximum variance and minimum loss of biological information. The eigengene levels are then inferred for the intermediate-risk group using the same weights obtained from PCA. It should be emphasized that regardless of which eigengenes are used, our network and the corresponding modules are consistently constructed based on both gene expression and DNA methylation data and they depend on the $\mu$ hyperparameter. The resulting eigengenes are robust features, carrying useful biological information, which can be leveraged in classification, clustering, and other downstream analyses including survival analysis.

### Survival analysis

To identify the optimal subset of modules for precise prognostication of risk groups, we conducted a two-step survival analysis using analyzeSurvival(). In the first step, we performed a penalized Cox regression analysis using the least absolute shrinkage and selection operator (lasso) penalty^29,30^ from the glmnet R package^61^ (Version 4.1.7). This analysis identified the three modules that were most associated with the survival data. Second, we fitted an AFT model^31^ to each combination of the top three modules and determined the optimal combination that leads to the smallest p-value. p-values were based on a log-rank test with a null hypothesis that there is no difference between the two high- and low-risk groups^62^.

To categorize the risk groups, iNETgrate uses findAliveCutoff() that searches for a cutoff on the AFT predictions such that the difference between high- vs. low-risk groups is optimized. More specifically, for each risk group, the function iterates over all possible cutoff values leading to a recall of more than a given threshold (i.e., for low-risk: minRecall=0.2, for high-risk: minRecall=0.1 in ROSMAP and 0.05 in other datasets) and selects the cutoff value that maximizes precision.

### Comparison with other prognostication approaches

To ensure the reliability of our integrative approach, we performed a comparative analysis by benchmarking our results against alternative methodologies including a well-known patient similarity network called SNFtool. We also compared our results vs. risk classification according to the clinical gold standards based on the intrinsic nature of the disease in each cohort.

### SNFtool

The SNFtool first computes a similarity matrix for each data type (i.e., gene expression and DNA methylation). That is, using each data type independently, a network is constructed where nodes are patients and weights of the edges represent similarity between patients computed based on correlation. The networks (similarity matrices) are then fused to create a consensus network representing the overall similarity between patients across different data types. The resulting patient similarity network is then used to cluster patients into subgroups. We noted that the SNFtool faced some limitations in using all the DNA methylation loci due to memory exhaustion while computing the similarity matrices. We tackled this issue by filtering out loci with a relatively low variation characterized by a standard deviation of less than 0.1. Determining the appropriate cutoff for a given dataset is subjective and challenging for SNFtool users.

### Clinical gold standards

In lung cohorts (LUSC and LUAD), we evaluated the risk groups based on the tumor stage. Specifically, we classified stages *I*, *IA*, *IB*, *II*, and *IIA* as the low-risk group, stages *IIIB* and *IV* as the high-risk group, and the remaining stages as the intermediate-risk group. In the LIHC cohort, we considered a case high-risk if the AFP level was greater than 500 or the Ishak fibrosis score was six. In contrast, patients were considered low-risk if their AFP levels were smaller than 250 and their Ishak fibrosis scores were 0, 1, or 2. The remaining cases were considered intermediate-risk. In the LAML cohort, we utilized the classification system available in the clinical data that categorized cases based on cytogenetic criteria into three groups of favorable (low-risk), intermediate, and poor (high-risk). We utilized the Braak score^63^ to stratify the ROSMAP cohort into three risk groups. Cases with a Braak score of 0, 1, or 2 were considered low-risk, those with a Braak score of 5 or 6 were classified high-risk, while the remaining cases were grouped as intermediate-risk.

### Supplementary Information

Supplementary Information.

## Data Availability

All data used in this study are publicly available. The cancer datasets can be accessed in The Cancer Genome Atlas (TCGA) at https://portal.gdc.cancer.gov/. The ROSMAP data is available from https://www.synapse.org/, with Synapse IDs syn3388564 (bulk RNA-seq) and syn5850422 (DNA methylation). Access to the ROSMAP data requires the submission of a Data Use Certificate through the AMP–AD website. The clinical data referenced in this study can be found in their respective publications.

## Code availability

iNETgrate is open-source and publicly available through Bioconductor (https://bioconductor.org/packages/iNETgrate/). We used Version 0.99.124 in this study.
