# Supplementary material for: Integrating DNA methylation and gene expression data in a single gene network using the iNETgrate package
Source: Sci Rep. 2023 Dec 8;13:21721. doi: 10.1038/s41598-023-48237-8 (PMC10709411; doi:10.1038/s41598-023-48237-8)
Supplement: Supplementary file 1 — Supplementary Information. [file 41598_2023_48237_MOESM1_ESM.pdf]

Integrating DNA methylation and gene expression data in a single gene network using the iNETgrate package

Sogand Sajedi[1,2,+], Ghazal Ebrahimi[3,+], Raheleh Roudi[4], Isha Mehta[5], Amirreza Heshmat[6], Hanie Samimi[7], Shiva Kazempour[1,2], Aamir Zainulabadeen[8], Thomas Roderick Docking[9], Sukeshi Patel Arora[10], Francisco Cigarroa[11], Sudha Seshadri[2,12,13], Aly Karsan[9,+], and Habil Zare[1,2,+,*]

[1]Department of Cell Systems & Anatomy, The University of Texas Health Science Center, San Antonio, Texas 78229, USA
[2]Glenn Biggs Institute for Alzheimer's & Neurodegenerative Diseases, San Antonio, Texas 78229, USA
[3]Bioinformatics Program, the University of British Columbia, Vancouver, BC, Canada
[4]Department of Radiology, Stanford University School of Medicine, Stanford, California 94305, USA
[5]Department of Immunology, University of Pittsburgh, Pittsburgh, Pennsylvania 15213, USA
[6]Department of Imaging Physics, The University of Texas MD Anderson Cancer Center, Houston, Texas 77030, USA
[7]School of Architecture, University of Utah, Salt Lake City, Utah 84112, USA
[8]Department of Computer Science, Princeton University, Princeton, New Jersey 08540, USA
[9]Canada's Michael Smith Genome Sciences Centre, British Columbia Cancer Research Centre, Vancouver, British Columbia, V5Z 1L3, Canada
[10]Mays Cancer Center, The University of Texas Health Science Center, San Antonio, Texas 78229, USA
[11]Malu and Carlos Alvarez Center for Transplantation, Hepatobiliary Surgery and Innovation, The University of Texas Health Science Center, San Antonio, Texas 78229, USA
[12]Department of Neurology, University of Texas, San Antonio, Texas 78229, USA
[13]Department of Neurology, Boston University School of Medicine, Boston, Massachusetts 02139,USA
[+]These authors contributed equally to this work.
[+]These senior authors contributed equally to this work.
[*]Corresponding author. Email: zare@uthscsa.edu. Mailing address: Department of Cell Systems & Anatomy, 7703 Floyd Curl Drive, San Antonio, TX 78229, USA. Phone:(210) 567-6797.

List of Supplementary Figures

List of Supplementary Tables

S1 iNETgrate models characteristics 2
S2 Number of patients 2

Supplementary Table S1. **Characteristics of the fitted iNETgrate model to each cohort**

Dataset	Best μ	Number of modules	Outliers	Best three eigengenes	Best combination	P–value
LUSC	0.4	71	3,971	23m, 44em, 64m	64m, 23m	10−7
LUAD	0.4	53	3,034	4e, 0e, 22e	4e, 22e	10−9
LIHC	0.0	19	8,185	2m, 8e, 19e	19e, 8e	10−9
LAML	0.0	25	4,758	10e, 19e, 24m	10e, 24m	10−6
ROSMAP	0.5	22	2,735	7em, 13em, 19e	19e, 7em	10−3

Supplementary Table S2. **The number of patients used in each step of analyses.**

Dataset	Total	Gene expression	DNA methylation	Survival data	iNETgrate network	SNF network	Survival analysis
LUSC	589	552	412	476	586	378	342
LUAD	592	574	490	483	589	475	417
LIHC	436	421	427	343	436	412	337
LAML	200	173	194	173	197	170	146
ROSMAP	3,586	638	740	3,583	840	538	538

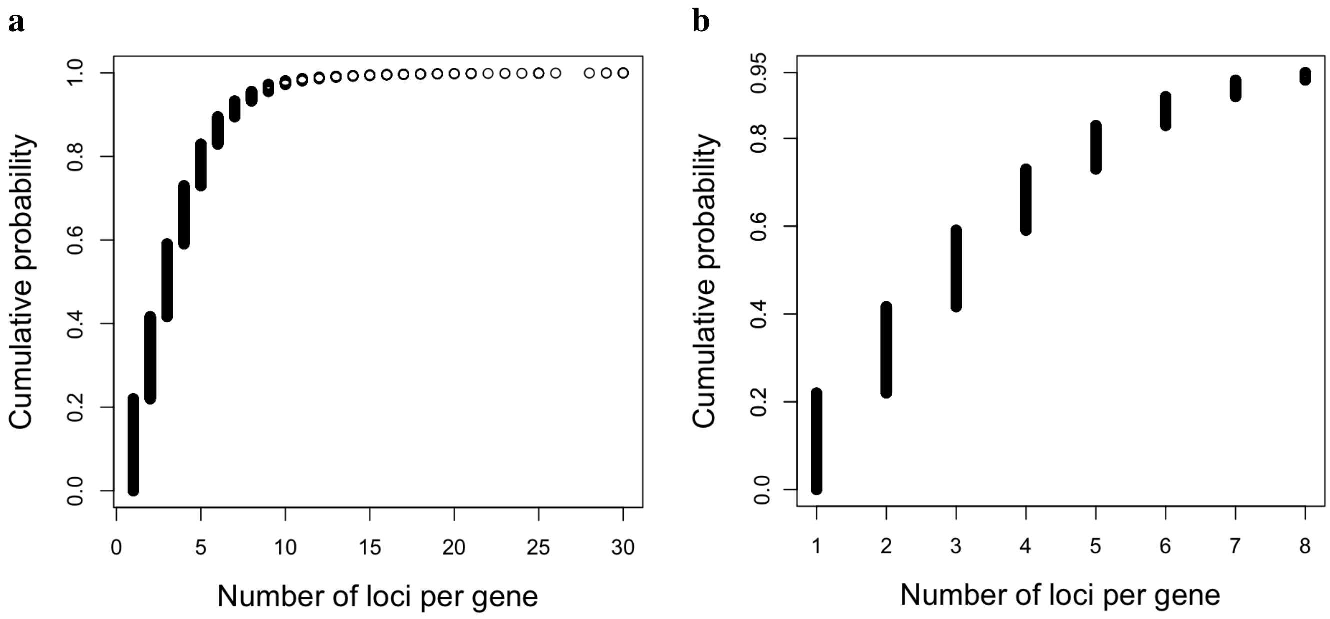

Supplementary Figure S1. The distribution of the number of loci per gene. The cumulative probability distribution of the loci per gene, excluding 103,036 loci with missing beta values in more than 50% of samples in the LUSC dataset. Out of the 382,541 remaining loci, 83,313 were not associated with any annotated gene resulting in 299,228 loci contributing to (**a**), which shows the number of loci per gene. (**b**) For 95% of these genes, the number is less than nine loci each.

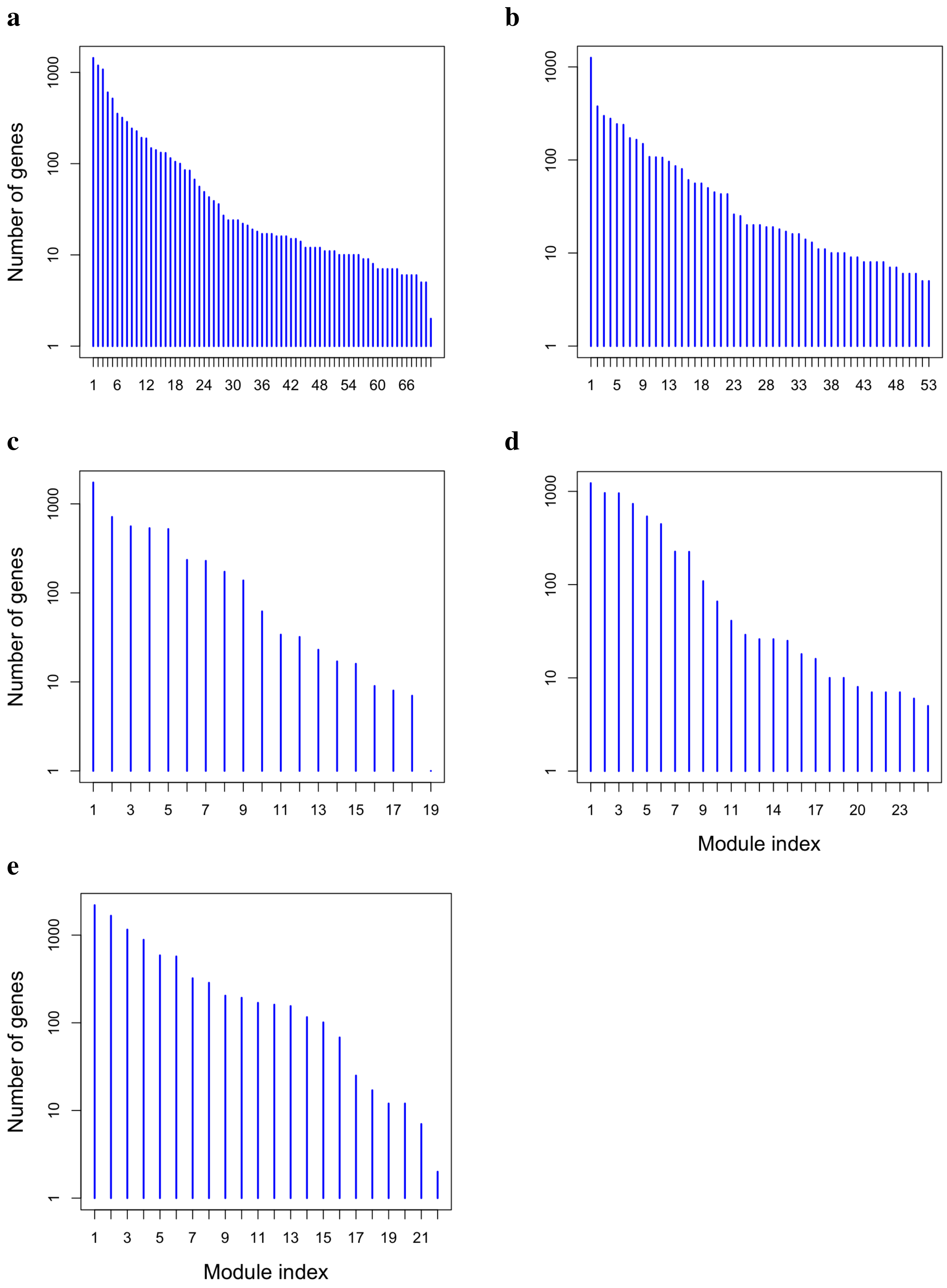

Supplementary Figure S2. The distribution of module sizes. The modules are sorted on the x-axes based on the number of genes in each module for (**a**) LUSC, (**b**) LUAD, (**c**) LIHC, (**d**) LAML, and (**e**) ROSMAP datasets.
